# Distinguishing Pediatric Lyme Arthritis of the Hip from Transient Synovitis and Acute Bacterial Septic Arthritis: A Systematic Review and Meta-analysis

**DOI:** 10.7759/cureus.2112

**Published:** 2018-01-25

**Authors:** Aristides I Cruz, Jr., Jason B Anari, Jose M Ramirez, Wudbhav N Sankar, Keith D Baldwin

**Affiliations:** 1 Orthopaedic Surgery, Hasbro Children's Hospital; 2 Orthopaedic Surgery, Children's Hospital of Philadelphia; 3 Orthopaedic Surgery, Warren Alpert Medical School of Brown University

**Keywords:** joint infection, limp, child, transient synovitis, septic arthritis, lyme arthritis

## Abstract

Objective

Lyme arthritis is an increasingly recognized clinical entity that often prompts orthopaedic evaluation in pediatric patients. While Lyme arthritis is most common in the knee, the clinical presentation of Lyme arthritis of the hip can be similar to both acute bacterial septic arthritis and transient synovitis. Accurately distinguishing these clinical entities is important since the definitive treatment of each is distinct. Because there is limited literature on monoarticular Lyme arthritis of the hip, the purpose of this study was to perform a systematic review and meta-analysis of clinical and laboratory parameters associated with Lyme arthritis (LA) of the hip and compare them to septic arthritis (SA) and transient synovitis (TS).

Study design

A systematic review of the literature was performed using the following search terms, including the variants and plural counterparts “hip” and “Lyme arthritis.” A final database of individual patients was assembled from the published literature and direct author correspondence, when available. A previously published cohort of patients with hip transient synovitis or septic arthritis was used for comparative analysis. A comparative statistical analysis was performed to the assembled database to assess differences in laboratory and clinical variables between the three diagnoses.

Results

Data on 88 patients diagnosed with Lyme arthritis of the hip was collected and consolidated from the 12 articles meeting inclusion criteria. The average age of patients presenting with Lyme arthritis was 7.5 years (± 3.5 years), the mean erythrocyte sedimentation rate (ESR), and the C-reactive protein (CRP) was 41 mm/hr and 3.9 mg/L, respectively. Peripheral white blood cell (WBC) count averaged 10.6 x 10^9^cells/L with the synovial WBC count averaging 55,888 cells/mm^3^. Compared to a previous cohort of patients with confirmed transient synovitis or septic arthritis, the 95% confidence interval for ESR was 21 - 33 mm/hr in those diagnosed with toxic synovitis (TS), 37 - 46 mm/hr for Lyme arthritis (LA), and 44 - 64 mm/hr for septic arthritis (SA). Synovial WBC counts (cells/mm^3^) 95% confidence intervals (CI) were 5,644 - 15,388 cells/mm^3 ^for TS, 47,533 - 64,242 cells/mm^3 ^for LA, and 105,432 - 260,214 cells/mm^3 ^for SA. There was a statistically significant difference in the incidence of fever > 38.5^o^C (P < 0.001) and refusal to bear weight (P < 0.01) between SA, LA, and TS.

Conclusions

Monoarticular Lyme arthritis can be a cause of hip pain in certain geographic areas and has clinical and diagnostic overlap with transient synovitis and acute bacterial septic arthritis. This study consolidates the available literature and represents the largest series of patients diagnosed with Lyme arthritis of the hip to date. We propose a diagnostic algorithm that serially incorporates ESR, followed by a synovial neutrophil count, when evaluating pediatric patients with an irritable hip in Lyme endemic areas.

## Introduction

Since its first description by Steere et al. in 1977 [1], Lyme disease has become the most common tick-borne illness in the United States and Europe [2-3]. Lyme disease is caused by the spirochete Borrelia burgdorferi and is transmitted by the Ixodes tick, which is endemic to certain areas of the United States, including the Northeast, parts of the upper Midwest, and the Pacific Northwest [2]. Reported cases of Lyme disease have a bimodal age distribution with average annual rates peaking in those between five to nine years old and 55-59 years old [4]. While Lyme disease can affect multiple organ systems, acute Lyme arthritis (LA) is an increasingly recognized clinical entity that prompts urgent or emergent evaluation in pediatric patients [4-9].

The most common joint affected by LA is the knee, and there have been multiple studies evaluating the diagnosis and treatment of this clinical entity in children [7, 10-14]. LA of the hip has also been described, albeit at a much lower population incidence compared to the knee [15-21]. Evaluation of a child with an acutely irritable hip continues to pose clinical and diagnostic challenges, particularly in geographic regions in which Lyme disease is endemic [17, 22-23]. The clinical presentation of LA of the hip can often be similar to both acute bacterial septic arthritis (SA) as well as transient synovitis (TS). In addition, there is no readily available, validated, and rapid point-of-care testing for Lyme disease. Therefore, accurately distinguishing these clinical entities is important, particularly because the definitive treatment of each is distinct. 

Because of the limited available literature on isolated LA of the hip, the primary purpose of this study was to perform a systematic review to consolidate all available studies examining pediatric patients with isolated LA of the hip. We also performed a meta-analysis of the clinical and laboratory parameters associated with LA of the hip compared to SA and TS. The goal of the meta-analysis was to assemble the data available in the literature and combine it to perform a comparative analysis in order to establish clinically useful diagnostic parameters distinguishing LA from bacterial SA or TS of the hip. 

## Materials and methods

We queried EMBASE, COCHRANE, and MEDLINE computerized literature databases from the earliest date available in the databases to August 7, 2017 using the following search terms (including variants and plural counterparts): “hip” and “Lyme arthritis.” A final database of individual patients was assembled from the published literature and direct author correspondence, when available. Reference lists from all articles were scrutinized to identify any additional articles of interest. Two authors (JBA, AIC) performed the initial search, while three authors (JBA, AIC, and JMR) independently reviewed the references of identified papers and selected those that fit the following criteria: (1) written in the English language, (2) level I, II, III, IV, and V study classification as detailed by “The Journal of Bone and Joint Surgery” criteria, (3) patient age ≤ 20 years old, and (4) individual patient level information available in the article or by direct correspondence with the original study author(s).

A previously published cohort of patients with hip transient synovitis or septic arthritis, examined at our home institution [15], was used for comparative analysis. In this cohort, SA was defined as either (1) a child with a positive synovial fluid culture for a bacterial pathogen, (2) synovial fluid with a white blood count (WBC) count > 50,000 cells/µL and positive blood culture results for a bacterial pathogen, or (3) frank purulence found at the time of surgery with subsequent negative cultures. The diagnosis of TS was one of exclusion, based on negative microbiology cultures and negative Lyme tests.

For comparative analysis, univariate P values were calculated by independent sample t-tests when variables were continuous, while Pearson’s Chi-squared and Fisher’s exact tests were used to assess differences in dichotomous, categorical outcome variables. Statistical significance was set at P < 0.05. Statistical analysis was performed using the Statistical Package for Social Sciences (SPSS), version 20.0 (IBM SPSS Statistics, Armonk, NY). 

## Results

**Figure 1:**
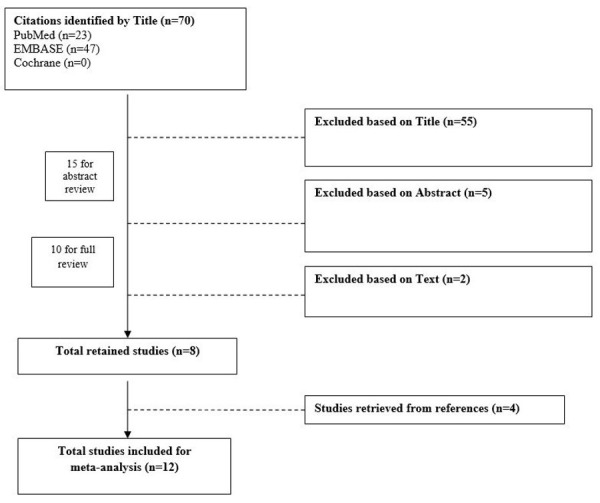
Flow Diagram Representing Systematic Review of Articles Included for Meta-analysis n: number

Data on 88 patients diagnosed with LA of the hip was collected and consolidated from the 12 articles meeting the inclusion criteria [6-7, 13-17, 19, 21, 24-26] (Figure 1, Table 1).

**Table 1:** Studies Included in Meta-Analysis

Author	Journal	Year	# Hip Lyme Patients
Bachur et al. [17]	Journal of Pediatrics	2015	20
Cruz et al. [15]	Journal of Pediatric Orthopedics	2017	17
Heyworth et al. [21]	Journal of Bone and Joint Surgery	2015	13
Milewski et al. [7]	Journal of Bone and Joint Surgery	2011	13
Glotbecker et al. [16]	Journal of Pediatric Orthopedics.	2011	8
Thompson et al. [13]	Pediatrics	2009	7
Amini et al. [24]	Pediatric Radiology	2007	2
Saulsbury [20]	Clinical Pediatrics (Phila)	2005	2
Willis et al. [14]	Journal of Pediatric Orthopedics	2003	2
Bachman et al. [6]	Pediatric Emergency Care	1998	2
Moak et al. [26]	Western Journal of Emergency Medicine	2012	1
Miller et al. [19]	Clinical Orthopaedics and Related Research	1993	1

There was a 2:1 predilection of males to females. Over 76% (67/88) of the patients were afebrile, defined as a temperature < 38.5^o^C. Two patients had a positive culture recorded (Table 2).

**Table 2:** Demographics and Clinical Variables of Patients Diagnosed with Hip Lyme Arthritis

Parameter		Count	Percentage
Sex	Male	39	44.3%
	Female	19	21.6%
	Not recorded	30	34.1%
Fever ≥ 38.5°C	Yes	20	22.7%
	No	67	76.1%
	Not recorded	1	1.1%
Refusal to bear weight	Yes	21	23.9%
	No	42	47.7%
	Not recorded	25	28.4%
Culture data	Not recorded	49	55.7%
	None taken	2	2.3%
	No growth	35	39.8%
	Culture positive	2	2.3%
Surgery	Yes	28	31.8%
	No	40	45.5%
	Not recorded	20	22.7%

One patient was positive for coagulase-negative S. aureus and the other was positive for a non-aureus Staphylococcus species. Table 3 summarizes the laboratory data for patients diagnosed with LA assembled from the available literature.

**Table 3:** Laboratory Data in Patients Diagnosed with Hip Lyme Arthritis WBC: white blood cell; ESR: erythrocyte sedimentation rate; CRP: C-reactive protein

Variable	# patients	Mean (SD; Range)
Age (years)	88	7.5 (3.5; 1.8 - 20)
Peripheral WBC count (x10^9^/L)	87	10.6 (3.2; 10.0 - 11.3)
ESR (mm/hr)	86	41 (20; 9 - 95)
CRP (mg/L)	70	3.9 (5.9; 0.1 - 40.2)
Synovial WBC (cells/mm^3^)	66	55,888 (33,985; 266 - 158,334)

The average age of patients presenting with Lyme arthritis was 7.5 years (standard deviation (SD): 3.5 years; range: 1.8 - 20 years), the mean and standard deviation for erythrocyte sedimentation rate (ESR) and C-reactive protein (CRP) was 41 ± 20 mm/hr and 3.9 ± 5.9 mg/L, respectively. Peripheral white blood cell (WBC) count averaged 10.6 x 10^9^cells/L (95% CI, 10.0 - 11.3) with the synovial WBC count averaging 55,888 cells/mm3 (95% CI, 47,533 - 64,242). The mean synovial polymorphonuclear cell percentage was 89% (95% CI, 86 - 92) in those for whom data were available.

Laboratory values for those diagnosed with LA were compared to a previous cohort of patients with confirmed TS or SA (Table 4) [15].

**Table 4:** Laboratory Data for Hip Lyme Arthritis vs. Septic Arthritis vs. Transient Synovitis LB 95% CI: Lower bound of 95% confidence interval; UB 95% CI: Upper bound of 95% confidence interval; TS: transient synovitis; LA: Lyme arthritis; SA: Septic arthritis; WBC: white blood cell; ESR: erythrocyte sedimentation rate; CRP: C-reactive protein; PMNs: polymorphonucleocytes

Parameter		# patients	Mean	LB 95% CI	UB 95% CI
Age (years)	TS	36	7.0	5.9	8.1
	LA	88	7.5	6.7	8.3
	SA	40	5.7	4.4	7.1
Peripheral WBC count (x10^9^/L)	TS	35	11.7	10.1	13.3
	LA	87	10.6	10.0	11.3
	SA	40	12.4	10.8	14.0
ESR (mm/hr)	TS	35	27	21	33
	LA	86	41	37	46
	SA	37	54	44	64
CRP (mg/L)	TS	26	4.7	2.7	6.7
	LA	70	3.9	2.5	5.3
	SA	36	8.8	6.6	10.9
Synovial WBC (cells/mm^3^)	TS	34	10,516	5,644	15,388
	LA	66	55,888	47,533	64,242
	SA	38	182,823	105,432	260,214
Synovial PMNs (%)	TS	28	63	52	74
	LA	20	89	86	92
	SA	37	88	84	92

**Figure 2:**
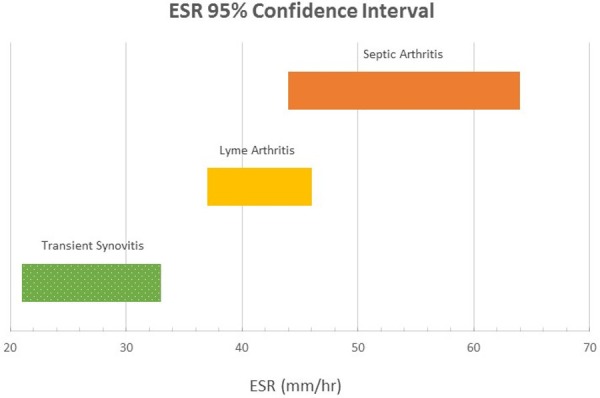
Erythrocyte Sedimentation Rate (ESR) 95% Confidence Intervals for Lyme Arthritis, Transient Synovitis, and Septic Arthritis

The 95% CI for ESR was 21 - 33 mm/hr in those diagnosed with toxic synovitis (TS), 37-46 mm/hr for LA, and 44 - 64 mm/hr for SA (Figure 2).

**Figure 3:**
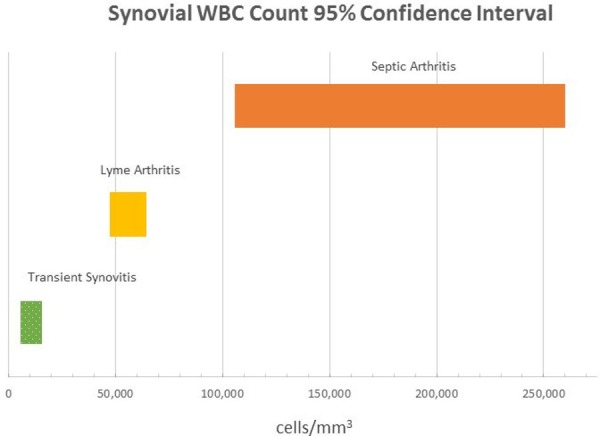
Synovial Fluid Neutrophil Count 95% Confidence Intervals for Lyme Arthritis, Transient Synovitis, and Septic Arthritis WBC: white blood count

Synovial WBC counts (cells/mm3) exhibited a similar trend. The 95% CIs were 5,644 - 15,388 cells/mm^3^ for TS, 47,533 - 64,242 cells/mm^3^ for LA, and 105,432 - 260,214 cells/mm^3^ for SA (Figure 3).

There was a statistically significant difference in the incidence of fever > 38.5^o^C (P < 0.001) (Table 5) and the refusal to bear weight (P = 0.01) (Table 6) between SA, LA, and TS.

**Table 5:** Presence of Fever > 38.5°C *P < 0.01 (Chi-squared test)

Diagnosis	Yes	No	% With Fever*
Transient Synovitis	11	25	30.5%
Lyme Arthritis	20	67	22.9%
Septic Arthritis	21	18	53.8%

**Table 6:** Refusal to Bear Weight *P < 0.01 (Chi-squared test)

Diagnosis	Yes	No	% Refusal to Bear Weight*
Transient Synovitis	22	14	61.1%
Lyme Arthritis	21	42	33.3%
Septic Arthritis	23	14	62.2%

## Discussion

Lyme arthritis of the hip is a relatively rare entity and accounts for 5 - 18% of cases of acute, inflammatory, non-traumatic hip pain in children [15, 17]. Differentiating between SA, LA, and TS of the hip can be challenging, even for the most discerning clinician. While Lyme arthritis of the knee has been well studied, monoarticular LA of the hip is less common; yet, it remains in the differential diagnosis when evaluating an irritable hip in a pediatric patient presenting in endemic areas. Because of this, the purpose of the current investigation was to synthesize the available literature of monoarticular LA in order to improve the clinician's ability to arrive at an accurate and timely diagnosis in patients presenting with an acutely painful hip.

This systematic review identified 88 patients diagnosed with monoarticular LA of the hip. We found that less than a quarter (22.9%) of patients with LA were febrile at presentation compared to more than half (53.8%) of those with SA. Additionally, we found a significant difference in the refusal to bear weight among patients with either SA or TS compared to those diagnosed with LA. Of patients in whom the ambulatory status was recorded, one-third (33.3%) of patients with LA refused to bear weight, while 62.2% and 61.1% of patients with SA and TS, respectively, refused to do so. This supports the authors’ clinical experience that LA of the hip seems to be associated with a less acutely irritable clinical presentation compared to either SA or TS.

In addition to history and physical examination, laboratory evaluation is often performed in patients who present with acute hip pain suspicious for an infectious or inflammatory etiology. Initial laboratory measurements typically include CBC, ESR, and CRP. In our analysis, patients with LA presented with a mean peripheral WBC count of 10.6 x 10^9^ cells/L (95% CI, 10.0 - 11.3) that was not significantly different than the mean serum WBC count in patients diagnosed with SA (12.4 x 10^9^ cells/L; 95% CI, 10.8 - 14.0) or TS (mean 11.7 x 10^9^ cells/L; 95% CI, 10.1 - 13.3). Therefore, our data suggests that peripheral WBC is not a reliable laboratory marker for distinguishing LA from TS or SA. 

With regards to ESR, we found that the LA group had ESR (mean: 41 mm/hr; 95% CI, 37 - 46) values that were not significantly different compared to those with SA (mean: 54 mm/hr; 95% CI, 44 - 64); however, compared to the those with TS (mean: 27 mm/hr; 95% CI, 21 - 32), patients with LA had significantly elevated ESR values. Moreover, we found that the range of ESR 95% confidence intervals ascended in an increasing order of diagnostic severity from TS --> LA --> SA (Figure 2). Our results show that while serum WBC count does not help distinguish LA from SA or TS, the ESR may help differentiate patients who would warrant further laboratory testing with either serum Lyme titers or intra-articular hip aspiration.

**Figure 4:**
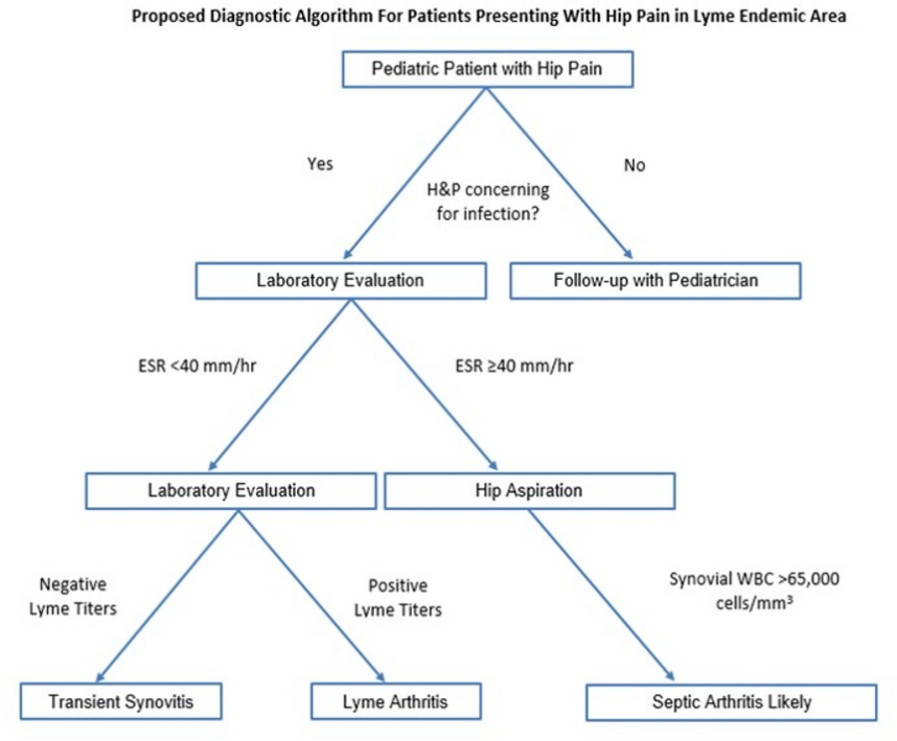
Proposed Diagnostic Algorithm for Pediatric Patients Presenting with Hip Pain in Lyme Endemic Area H&P: history and physical; ESR: erythrocyte sedimentation rate; WBC: white blood count

Based on our data, we propose a diagnostic algorithm in which an ESR level ≥ 40 mm/hr warrants a hip aspiration to rule out SA in the absence of other symptoms or clinical parameters suspicious for the latter (Figure 4).

In other words, in a patient with an equivocal history, physical exam, and laboratory values, an ESR ≥ 40 should prompt the clinician to obtain hip synovial fluid to evaluate for SA. An ESR level < 40 mm/hr could be worked up further with Lyme serology as LA or TS become more likely. Of course, if significant hip irritability or other clinical signs of SA exist, then treatment for SA should be initiated. The authors stress that this proposed algorithm should be used merely as a guide to assist with clinical decision making and it is at the treating physician’s discretion as to the most appropriate diagnostic and treatment course. Figures 2-3 describe potential guideline numbers for ESR and peripheral WBC count when assessing a patient with an irritable hip. The figures illustrate the concept of these three diagnoses existing on a continuum of inflammation, with TS being the least inflammatory and SA the most inflammatory. This clinical picture is consistent with basic science literature supporting the idea that intra-articular neutrophils associated with SA are inherently different from those associated with other inflammatory etiologies [27-28], which seems to be consistent with the results of the present study. 

After the aforementioned ESR level, the algorithm presented herein utilized a synovial neutrophil count threshold to assist the clinician in the decision to perform surgical drainage of the hip. As seen with ESR, the 95% confidence intervals for synovial WBC counts also increased with increasing disease severity. Moreover, there was no observed overlap in the 95% confidence intervals between LA and SA (Figure 3). Thus, we propose utilizing the upper end of the 95% confidence interval (65,000 cells/mm^3^) as a potential cutoff, with values above this number strongly suggestive of bacterial SA. Utilization of gram stain results should also be taken into account; however, the negative predictive value of gram stain results in the evaluation of acute SA limits its utility [29-30].

Limitations   

There are several limitations to this study. This was a systematic review of previously published articles which were retrospective investigations in and of themselves. Therefore, our study was subject to the limitations inherent in retrospective studies. When selecting studies to include in our meta-analysis, we were stringent in our criteria of having patient-level data available from prior studies in order to help determine (as best as possible from the available literature) clinically relevant parameters that may help clinicians distinguish the three different disease processes investigated. When not available from the published manuscript, we made every effort to contact the original study authors to gain access to the raw data from the primary investigation. 

Because of the relatively low reported incidence of isolated LA of the hip, our methods allowed us to pool data from a variety of studies and make clinically meaningful comparisons between LA, SA, and TS. This study must be taken in context, however, as one must be cautious when pooling laboratory and clinical data from heterogeneous sources. Different laboratories may have different methods and different investigators may have defined clinical parameters, such as weight-bearing, differently. In addition to heterogeneous sources, there may have also been some bias introduced in this systematic review since three out of the 12 reviewed articles were reported from the same institution [16-17, 21]. This may have inflated the true incidence of LA in the studied sample. We also used a historical cohort for our comparative analyses, and these patients may be inherently different from those pooled together from the literature. We chose to do this primarily because the studies included in our systematic review lacked adequate comparison groups and these historical controls were evaluated at our home institution, which should help limit any variability in data collection. Finally, we must emphasize that the proposed diagnostic algorithm, as described, has not been prospectively validated and is based primarily on the authors’ experience and the findings of the current systematic review. Ideally, multicenter prospective studies or retrospective reviews of prospectively collected data, such as registries, would help counter the limitations of our investigation. 

## Conclusions

Monoarticular LA can be a cause of hip pain in certain geographic areas and has a clinical and diagnostic overlap with TS and acute bacterial SA. This study consolidates the available literature and represents the largest series of patients diagnosed with LA of the hip to date. We propose a diagnostic algorithm that serially incorporates ESR followed by synovial neutrophil count when evaluating pediatric patients with an irritable hip in Lyme endemic areas. 
